# An integrated parity-time symmetric wavelength-tunable single-mode microring laser

**DOI:** 10.1038/ncomms15389

**Published:** 2017-05-12

**Authors:** Weilin Liu, Ming Li, Robert S. Guzzon, Erik J. Norberg, John S. Parker, Mingzhi Lu, Larry A. Coldren, Jianping Yao

**Affiliations:** 1 Microwave Photonics Research Laboratory, University of Ottawa, 25 Templeton Street, Ottawa, Ontario, Canada K1N 6N5; 2 Department of Electrical and Computer Engineering, University of California Santa Barbara, Santa Barbara, California 93116, USA

## Abstract

Mode control in a laser cavity is critical for a stable single-mode operation of a ring laser. In this study we propose and experimentally demonstrate an electrically pumped parity-time (PT)-symmetric microring laser with precise mode control, to achieve wavelength-tunable single-mode lasing with an improved mode suppression ratio. The proposed PT-symmetric laser is implemented based on a photonic integrated circuit consisting of two mutually coupled active microring resonators. By incorporating multiple semiconductor optical amplifiers in the microring resonators, the PT-symmetry condition can be achieved by a precise manipulation of the interplay between the gain and loss in the two microring resonators, and the incorporation of phase modulators in the microring resonators enables continuous wavelength tuning. Single-mode lasing at 1,554.148 nm with a sidemode suppression ratio exceeding 36 dB is demonstrated and the lasing wavelength is continuously tunable from 1,553.800 to 1,554.020 nm.

## Abstract (web summary)

The breaking of parity-time symmetric gain and loss profiles can be used to achieve single-mode lasing in coupled microring resonators. Here, Liu *et al*. show that this effect can be electrically controlled with a tunable lasing wavelength and strong sidemode suppression.

Mode management in a laser cavity is an important topic in laser physics and has been extensively investigated^1^,^2^. Owing to the broad gain bandwidth of a laser cavity, mode management is required to select the desired mode and to suppress other modes to achieve single-mode operation, which is required for applications such as in optical communications systems^2^ and nonlinear optical systems^3^. At present, there are four major approaches that have been extensively studied for mode management in a laser cavity. The first approach is to use optical feedback in a cavity to achieve single-mode operation^4^,^5^,^6^; the second is to enhance mode confinement by reducing the mode size in a laser cavity to achieve single-mode operation^7^,^8^,^9^; the third is to shape the spatial profile of a pump light to a laser cavity to achieve mode selection^10^; and the fourth approach is to use parity-time (PT) symmetry^11^,^12^,^13^,^14^,^15^,^16^ to implement mode selection. The last approach has been an active topic and has been heavily researched recently^17^,^18^,^19^,^20^,^21^.

Specifically, in the first approach, an optical cavity is incorporated into the active region of a laser structure for mode selection^4^,^5^. A strong feedback for a mode that is determined by the optical cavity would lead to a strong optical oscillation or lasing at that mode. The optical feedback can be achieved, for example, using an ultrashort cavity implemented by a pair of reflective mirrors^4^ or a distributed feedback (DFB) grating^5^. An ultrashort cavity (∼5.5 μm) with a large free spectral range (FSR) offers strong mode selectivity for single-mode lasing^4^. A DFB laser has an active region containing a periodically structured grating to provide a strong optical feedback for a single longitudinal mode operation, in which tunable operating wavelength is possible by thermal tuning^6^. However, the cavity feedback structure in a DFB laser is complicated and the use of such a structure would increase the fabrication complexity.

In the second approach, a metallic cavity is used to reduce mode size and enhance mode confinement for mode selection based on plasmonics. In a metallic cavity, surface plasmon polaritons excited at the metal-dielectric interfaces can provide an extremely strong light confinement, which enable intense, coherent and directional optical emission that is below the diffraction barrier^7^,^8^. With light waves confined in a volume structure in subwavelength dimensions, plasmon lasers can have a very small footprint on the nanoscale. However, very high gain is needed to enable lasing due to very high losses in metals^9^.

In the third approach, lasing mode selection is achieved by shaping the spatial profile of the optical pump to the laser cavity. In a laser cavity, possible high-*Q* lasing modes exhibit distinct emission patterns, which can be selected by adaptively controlling the spatial profile of the pump light to achieve single-mode lasing^10^. To select a desired mode while suppressing other modes, the optical pump with a specific spatial profile is needed. The spatial profile of the optical pump for a desired lasing mode can be obtained by a genetic algorithm and specific optical pump can be realized by using a spatial light modulator^10^. This approach provides flexible mode selection but a time-consuming genetic algorithm is needed to search for the optimum pump profile for a desired lasing mode.

In the fourth approach, mode selection is achieved based on PT symmetry by manipulating the interplay between gain and loss in a laser cavity^17^,^18^,^19^,^20^,^21^. In a coupled arrangement with two identical microring resonators one is experiencing gain, whereas the other is experiencing an equal-magnitude loss, to form a PT-symmetry situation. By changing the relationship between the gain and loss, and the coupling between the two microring resonators, one can selectively break the PT-symmetry condition for a desired mode, which can be used to improve the maximum achievable gain for this mode. Therefore, the desired mode can be controlled for single-mode operation in an inherently multi-mode microring laser^18^. The breaking of the PT-symmetry condition provides a simple and effective solution to achieve single-mode lasing by allowing the desired mode to have a higher gain, while suppressing other modes^17^,^18^,^19^,^20^,^21^. In refs 18, 19, a single-mode lasing was demonstrated with an enhanced mode discrimination by using the exceptional points in a PT-symmetric coupled ring resonator structure^20^. However, the lasing wavelength was not tunable and the sidemode suppression ratio was only 21 dB.

In this study, we propose and experimentally demonstrate an electrically pumped integrated microring laser that supports single-mode operation based on PT symmetry with an improved mode suppression ratio. The microring laser has a coupled arrangement in which two structurally identical microring resonators are mutually coupled via a tunable coupler, to enable truly PT-symmetric operation. By incorporating two semiconductor optical amplifiers (SOAs) in each of the two microring resonators, the gain–loss can be controlled by changing the injection currents to the SOAs. As the coupling between the two microring resonators is achieved by a tunable coupler, the coupling coefficient can be precisely controlled, to maintain or break the PT-symmetry condition for mode selection. In addition, the resonance wavelength can be controlled by changing the injection currents to the phase modulators (PMs) in the two microrings. Thus, the proposed PT-symmetric single-mode laser has the following advantages. First, compared with the biased PT-symmetric system in ref. 18, the proposed coupled ring system is truly PT symmetric. Second, the proposed laser is electrically pumped, whereas in refs 17, 18 the lasers were optically pumped. Finally, the operating wavelength of the proposed laser can be continuously tuned by tuning the injection current to the PM in the ring resonator, which can also be used for compensating the phase mismatch between the two ring resonators almost in real time. Compared with a previously reported mode suppression ratio of 21 dB in a PT-symmetric single-mode laser^18^, the proposed laser presents an increased mode suppression ratio of 36.07 dB and the lasing wavelength is electronically tunable with a tuning range of 0.22 nm.

## Results

### Basic principle

**Figure 1:**
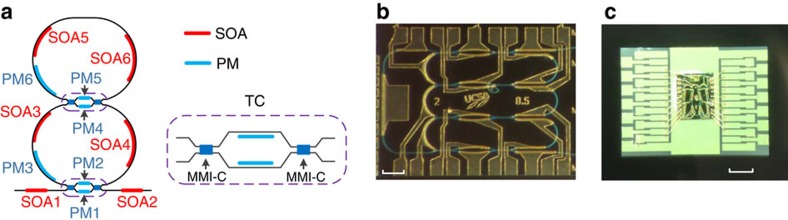
The schematics of the proposed single-mode microring laser. (**a**) The schematic diagram of the microring laser consisting of two coupled rings and a bypass waveguide. (**b**) A photograph of the fabricated microring laser prototype with a scale bar of 200 μm. (**c**) A photograph of the laser wire bonded to a customized carrier for experimental test with a scale bar of 1 mm. MMI-C, multimode interference coupler; PM, phase modulator; SOA, semiconductor optical amplifier; TC, tunable coupler.

The schematic of the proposed wavelength-tunable single-mode microring laser is shown in Fig. 1a. It consists of two structurally identical ring resonators that are mutually coupled by a tunable coupler. Within each microring resonator, there are two SOAs to enable gain control and a PM to enable wavelength tunability. By changing the injection currents to the SOAs, the gain or loss in each microring resonator can be controlled. A bus waveguide is also coupled to the bottom ring resonator for lasing output. The tunable coupling between the two coupled microring resonators and between the bottom ring and the bus waveguide is realized using two tunable couplers, each consisting of two multi-mode interference (MMI) couplers and two PMs, as shown in the inset in Fig. 1a. The coupling ratio of each tunable coupler can be continuously tuned by adjusting the injection currents to the two PMs. Under the PT-symmetry condition, the gain and loss are identical in the two microring resonators^18^, which can be achieved by controlling the injection currents to the SOAs in the two ring resonators. In this case, a mode with a gain smaller than the total coupling coefficient of the two ring resonators will not lase. If the gain exceeds the total coupling coefficient, the PT-symmetry condition will be broken and a lasing mode will appear. With such a mode selection mechanism, the cavity resonance modes in a coupled ring resonator can be efficiently controlled without the need of additional filters.

In the time domain, the interplay between the *n*th longitudinal modes in the two microrings obeys two coupled differential equations for their respective modal amplitudes, *a*_*n*_, *b*_*n*_ (ref. 12), given by


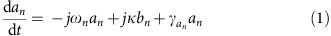



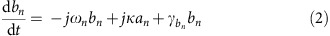


where 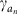 and 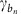 represent the net gain or loss in each microring resonator, *ω*_*n*_ is the angular frequency of the *n*th longitudinal mode and *κ* is the coupling coefficient between the two microring resonators. According to equations (1) and (2), the eigen frequencies, 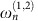, of the two supermodes of this system are given by


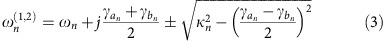


In the PT-symmetry situation, we have 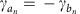 and equation (3) is simplified to


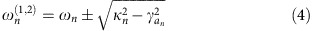


It can be seen from equation (4) that any pair of modes whose gain/loss remains below the coupling coefficient 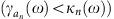 will remain neutral. However, as soon as the gain/loss exceeds the coupling coefficient 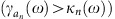, the PT-symmetry condition will be broken and a conjugate pair of lasing or decaying modes will emerge.

### Device design

As ring resonators are used in the design, low-radius waveguide bends with a low bend radiation loss are required. A deeply etched waveguide geometry is used due to its high optical confinement, which can reduce bending losses in a small-radius waveguide bend. Typically, it is desired that a waveguide in a photonic integrated circuit (PIC) supports only a single mode. In the transverse direction, the waveguide is able to support a single-mode by the proper epitaxial structure design. In the lateral direction, the number of modes is defined by the width of the waveguide. Owing to the highly confined nature of the deeply etched geometry, the cutoff for the first odd mode is at a very narrow width of about 1.1 μm. However, a narrow deeply etched waveguide has a high scattering loss and potentially high surface recombination current compared with a wider waveguide. For this reason, in our PIC, wider multi-mode waveguides were used. In particular, 2.8 μm-wide waveguides were used in the active, passive and PM propagation regions, and were tapered down to 1.8 μm at the inputs and outputs of the MMI couplers. This narrower waveguide allowed us to design short (100 μm) couplers. Despite the waveguide of both widths supporting multiple modes, the PIC operates in a single-mode manner, because the components in the PIC favour the fundamental mode. Higher-order modes have higher scattering loss over the fundamental mode. This is due to an increased optical power at the edges of the waveguide. In the active regions, the first odd mode has a reduced gain when compared with the fundamental mode, because the current density at the edges of the waveguide is lower due to surface recombination. This is where the optical intensity is the highest for the first odd mode. Most importantly, MMI couplers are designed to be low loss for the fundamental mode. Owing to the decreased effective index, MMIs are highly lossy to high-order modes. For example, the MMI couplers theoretically show a 5 dB suppression for the first odd mode. This mode-filtering is crucial to achieve single-mode operation of the PIC. In fact, no multi-mode effects were witnessed in the fabricated PIC.

**Figure 2:**
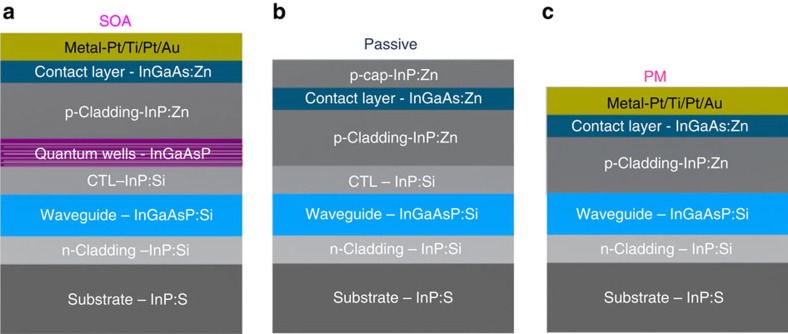
The epitaxial structures of the components in the proposed microring laser. (**a**) The epitaxial structure of the SOA region, which has five QWs above the CTL. (**b**) The epitaxial structure of the passive waveguides without metal contacts, which are used for low-loss passive waveguide propagation sections. (**c**) The epitaxial structure of the phase modulator (PM) region, in which the CTL is removed to provide efficient current injection into the waveguide for high efficient phase tuning. Layer thickness: 150 nm contact layer, 1.7 μm p-cladding, 0–250 nm CTL, 300 nm waveguide and the QW layer contains 65 Å QWs and 80 Å barriers.

A prototype of the proposed microring laser is fabricated in an InP-InGaAsP material system, as shown in Fig. 1b, which is also wire bonded to a carrier for experimental demonstration, as shown in Fig. 1c. In the prototype, the length of the deeply etched waveguide ring is 3 mm. Two 400 μm SOAs with a confinement tuning layer (CTL) offset quantum well (QW) structure^22^ are fabricated in the microring, to provide a peak gain of 9.6 dB per SOA. The epitaxial structure for the passive and active regions in the device is illustrated in Fig. 2 and discussed in Methods. With 3 mm of ring length and 1.7 cm^−1^ of passive waveguide loss, the total waveguide propagation loss is 1.6 dB. For a ring with 10% cross-coupling and 0.5 dB MMI insertion loss, the couplers add about 2 dB of loss. Thus, the total round-trip loss is 3.6 dB, which is compensated for by the two SOAs. Two additional active SOAs are incorporated in both input and output waveguides, to compensate for the fibre coupling losses. In addition, the waveguides are angled at 7° to minimize the reflections. Phase modulation in the ring and the tunable MMI Mach-Zehnder interferometer (MZI) coupler is accomplished by a forward bias current, to introduce free carrier absorption through the carrier plasma effect. A PM in the chip has a standard length of 300 μm.

### Tunable coupler and SOA characterization

**Figure 3:**
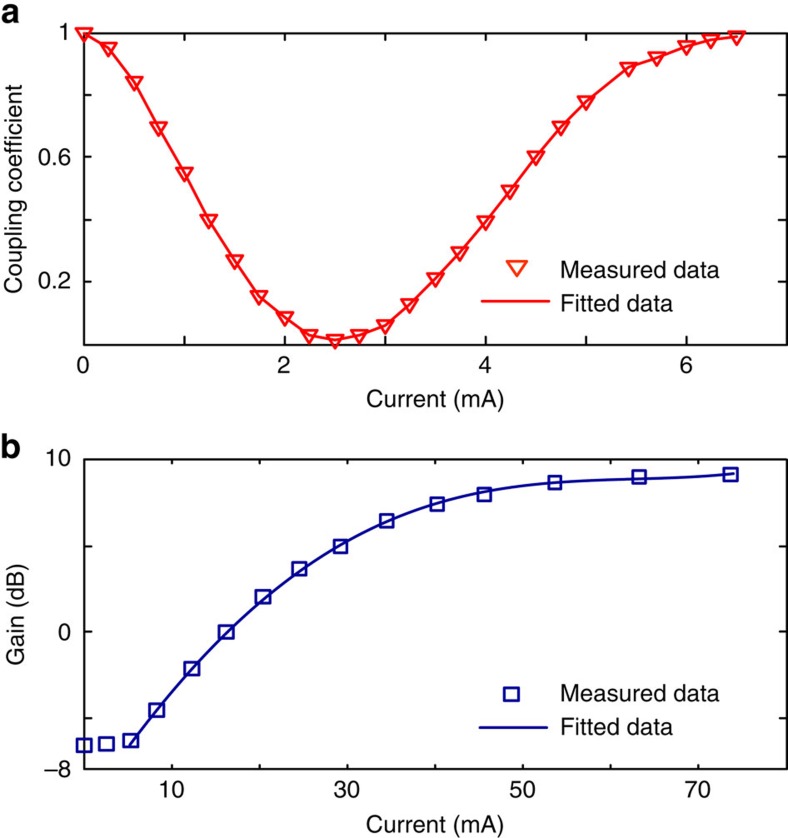
Experimental results to show the component performance in the proposed laser. (**a**) Tunable coupling coefficients of an multimode interference Mach–Zehnder interferometer coupler at different injection currents ranging from 0 to 6.5 mA, to one PM in one of the two arms. A nonlinear cosine fitting is used in the fitted data. (**b**) The gain profile of a semiconductor optical optical amplifier as a function of the injection current from 0 to 75 mA. A quartic polynomial fitting is used in the fitted data.

The coupling coefficient of a tunable coupler is measured at a different injection current to one of the two PMs, which can be controlled from 0 to 100% when the PM is injected with a current from 0 to 2.5 mA. Figure 3a shows the measured coupling coefficient as a function of the injection current to the PM on the upper arm of the MMI Mach-Zehnder interferometer coupler, from 0 to 6.5 mA. The large signal gain profile of an SOA is also measured. The SOA has a maximum gain of 9.6 dB when the injection current is above 70 mA, as shown in Fig. 3b.

### Single-mode lasing experiment

**Figure 4:**
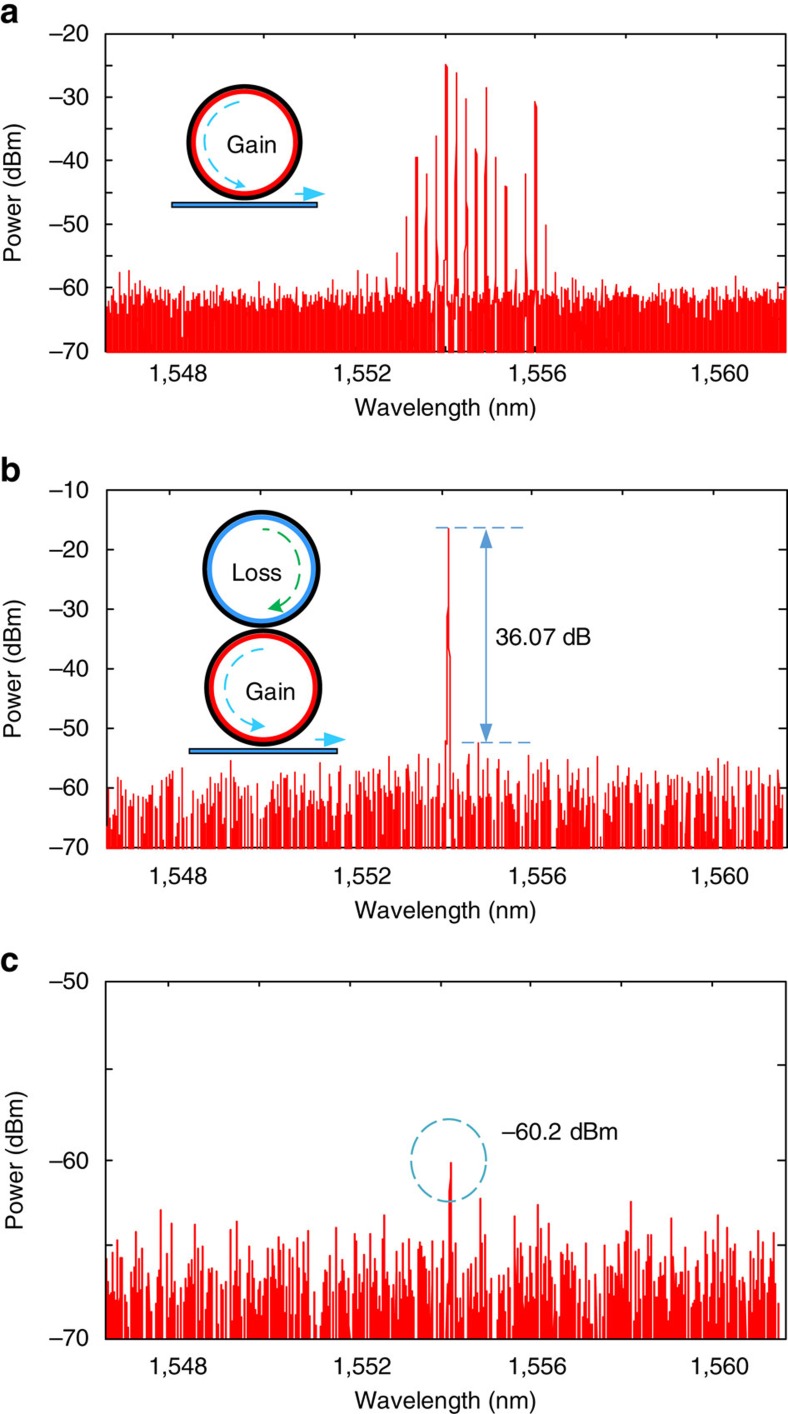
Experimental results to show mode suppression by breaking the PT-symmetry condition. (**a**) Emission spectrum of a single ring resonator when the two SOAs inside the microring resonator are driven by an injection current of 21 mA each. (**b**) Single-mode spectrum under the PT-symmetry condition. The SOAs in the upper ring resonator are not activated, and the SOAs in the lower ring resonator are driven by an injection current of 21 mA each. The mode suppression ratio is 36.07 dB. (**c**) The optical spectrum at the other output of the laser showing the counter propagating modes and the counter propagating mode at the lasing frequency is marked with a dashed circle.

**Figure 5:**
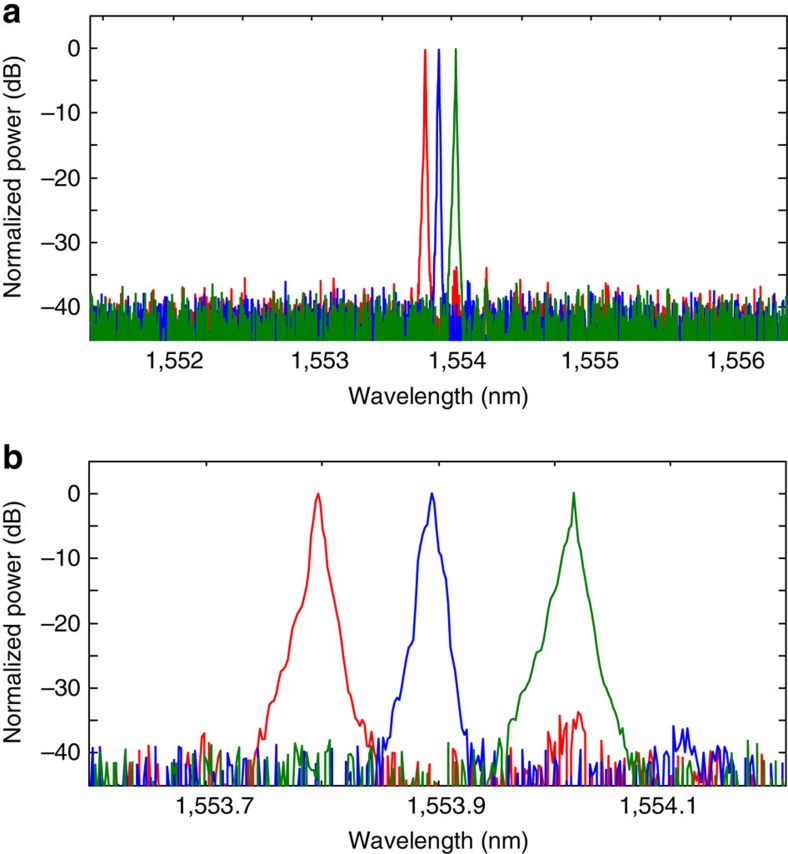
Experimental results to show tunable single-mode lasing under PT-symmetry condition. (**a**) The wavelength is tuned to three different values by applying three different current pairs to phase modulator PM3 (1, 3 and 5 mA) and PM6 (1.1, 3.1 and 5.2 mA) in the ring resonators. (**b**) A zoom-in view of the wavelength tuning.

An experiment to validate the single-mode lasing in the proposed microring laser based on PT symmetry is implemented. By changing the injection currents to the SOAs in the two microring resonators and the injection currents to the tunable couplers, the gain/loss and coupling coefficients can be tuned precisely to satisfy the PT-symmetry condition^18^. As shown in Fig. 4a, an emission spectrum with multiple modes in a single microring resonator is observed when the cavity gain exceeds the loss. Once the PT symmetry is established by tuning the gain/loss and the coupling coefficients in the two ring resonators, single-mode lasing with a wavelength at 1,554.148 nm occurs, as shown in Fig. 4b, where the injection currents to the active components are given in Table 1. The light from the PT-symmetric laser is coupled out of the chip using a lensed fibre and the optical power at the output of the lensed fibre is measured to be −14.0 dBm. Considering that the coupling loss between the lensed fibre and the waveguide is 12.7 dB, the optical power directly from the chip is −1.3 dBm. The presence of the lossy ring serves to suppress the unwanted modes with a side mode suppression ratio exceeding 36 dB, due to the tunable gain, loss and coupling efficiency. The counter propagating modes are also measured and shown in Fig. 4c. It can be seen that the counter propagating mode at the lasing frequency has a power of −60.2 dBm, which is 46.2 dB less than the lasing mode, which is mainly due to the reflection at the output facet. By changing the injection currents to the PMs in the two ring resonators, the lasing wavelength can be continuously tuned. In the experiment, a wavelength tuning range from 1,553.800 to 1,554.020 nm is achieved, as shown in Fig. 5, which is equal to the FSR of the microring resonator. For a ring resonator of a length of 3 mm, the FSR is 0.22 nm. The modal discrimination of the PT-symmetric laser is also measured, which is 13.19 dB (Supplementary Methods). As a comparison, the modal discrimination of a conventional ring laser implemented using the same PIC is also measured, which is 3.26 dB. Thus, an increase in modal discrimination of 9.93 dB is achieved.

## Discussion

In the experiment, the total power consumption of the microring laser is 205.1 mW, including 52.6 mW consumed by the output SOAs (SOA2), which can be avoided in a packaged laser without a large fibre coupling loss. In this case, the total power consumption for such a PT-symmetry microring laser can be reduced to 152.5 mW. For real applications, a single SOA with a peak gain of 9.6 dB in a ring resonator is enough to compensate for the total roundtrip loss. As shown in the gain profile of the 400 μm SOA in Fig. 3b, an injection current of ∼27 mA can provide a gain of 4.5 dB, which is large enough to ensure the microring resonator to operate under the same condition, as shown in Table 1. As a result, the total power consumption can be further reduced to 126 mW.

In conclusion, we have proposed and experimentally demonstrated a photonic integrated PT-symmetric single-mode microring laser based on two mutually coupled active microring resonators. Thanks to the tunable gain or loss in the microring resonators and the tunable coupling coefficient in the tunable coupler, single-mode operation with a large mode suppression ratio and a continuously tunable wavelength range of 0.22 nm was demonstrated. The two mutually coupled microring resonators in the microring laser were implemented based on InP-InGaAsP with each resonator having two SOAs and a PM incorporated. The incorporation of the SOAs in the ring resonators ensures a precise electrical control of the interplay between gain and loss to achieve PT-symmetry condition and incorporation of the PMs in the ring resonators enables wavelength tuning. By tuning the gain and loss in the two microring resonators to achieve the PT-symmetry condition, a single-mode lasing at 1,554.148 nm with a sidemode suppression ratio exceeding 36 dB was demonstrated. By adjusting the injection currents to the PMs, the lasing wavelength was continuously tuned from 1,553.800 to 1,554.020 nm with a tuning range of 0.22 nm.

## Methods

### Device epitaxial structure

The device is fabricated in the InP-InGaAsP material system. An n-doped layer is grown on top of the InP substrate and a waveguide layer is then grown on top of the n-doped layer, which has a thickness of 300 nm, on top of which there is a CTL with a thickness of ∼250 nm. For an SOA, there are five QWs grown on top of the CTL, which pushes the QWs away from the waveguide layer to reduce the confinement factor and improve the saturation power. The QW layer is covered by a Zn p-doped layer with a thickness of 1.7 μm. For a passive waveguide, the CTL is covered by the p-doped layer without the QWs. For a PM, the p-doped layer is grown on top of the waveguide layer without the CTL and the QWs. For both the active and passive regions, there is a 150 nm contact layer on top of the p-doped layer and the contact layer is covered by a p-cap layer for the passive waveguides and by a metal layer for the active regions.

### SOA gain profile measurement

The gain profile is measured by using a 400 μm standalone SOA with the same design as the SOAs in the ring resonators, and the lensed fibre to waveguide coupling loss and on chip waveguide loss are also measured to calibrate the gain profile. A continuous wave light wave at 1,554.148 nm generated by a tunable laser source is coupled into the standalone SOA by a lensed fibre. The output power of the SOA is measured at different injection currents by an optical spectrum analyser and the measured gain profile is fitted by a quartic polynomial. As the gain profile for a QW SOA is temperature dependent, the internal temperature of a SOA under normal operation is >60 °C without external temperature control; thus, the efficiency of the SOA is reduced due to a high temperature. In addition, the alignment between the lensed fibre and the on-chip waveguide where the measured SOA is located can be shifted without temperature control due to heat accumulation in the measured SOA, which will add loss to the measurement data.

### Data availability

All data are available from the corresponding author upon reasonable request.

## Additional information

**How to cite this article:** Liu, W. *et al*. An integrated parity-time symmetric wavelength-tunable single-mode microring laser. *Nat. Commun.* **8,** 15389 doi: 10.1038/ncomms15389 (2017).

**Publisher's note**: Springer Nature remains neutral with regard to jurisdictional claims in published maps and institutional affiliations.

## Supplementary Material

Supplementary Information Supplementary Methods, Supplementary Tables and Supplementary References

## Figures and Tables

**Table 1:** The injection currents to the SOAs and PMs.

**Component**	**Injection current**	**Gain**
SOA1	0	0
SOA2	25.000 mA	∼3.7 dB
SOA3	21.422 mA	∼2.3 dB
SOA4	21.051 mA	∼2.2 dB
SOA5	17.513 mA	∼0.6 dB
SOA6	17.397 mA	∼0.5 dB
PM1	0	N/A
PM2	2.011 mA	N/A
PM3	0	N/A
PM4	0	N/A
PM5	1.282 mA	N/A
PM6	0.103 mA	N/A

NA, not applicable; PM, phase modulator; SOA, semiconductor optical amplifier.
