# Supplementary material for: An integrated parity-time symmetric wavelength-tunable single-mode microring laser
Source: Nat Commun. 2017 May 12;8:15389. doi: 10.1038/ncomms15389 (PMC5437294; doi:10.1038/ncomms15389)
Supplement: Supplementary Information — Supplementary Methods, Supplementary Tables and Supplementary References [file ncomms15389-s1.pdf]

Supplementary Methods

In a single-ring laser, all longitudinal modes with positive net gain are allowed to lase. To achieve single mode lasing, the net gain is controlled to allow one mode with a positive net gain. Without an extra cavity or filter to select a desired mode, two adjacent modes may have a close or even an identical net gain which would limit the modal discrimination. However, a parity-time (PT) symmetric laser would enhance the gain-loss contrast between modes [1]. In a single mode PT-symmetric laser, the gain and loss would balance each other by tuning the gain, loss, and the coupling between the two coupled ring resonators. Above this threshold, PT-symmetry breaking would occur, in which case the fundamental mode splits into two modes: one experiencing a gain while the other experiences a loss [2]. In this way, the modal discrimination is enhanced. An example to show a stronger modal discrimination in a PT laser than that in a conventional laser can also be found in [3].

The modal discrimination of a laser is the round-trip loss ratio between the fundamental mode and the next higher order mode [4]. To measure the modal discrimination, the lasing conditions for single and dual mode operation of the proposed PT-symmetric laser and a single ring laser are recorded, which are the gains in dB of the semiconductor optical amplifiers (SOAs). The modal discrimination is then extracted from the gain contrast between the single mode and the dual-mode lasing conditions.

The SOA gain profile shown in Fig. 3b in the manuscript is a small-signal gain, and a gain under saturated conditions should be employed when measuring the modal discrimination. In a multiple quantum-well SOA, gain saturation results from the depletion of carrier density in the active region due to high levels of stimulated recombination. Though the carriers are depleted in accordance with the wavelength of the light, fast intraband scattering equalizes the carrier density such that the semiconductor gain saturates homogeneously across the entire gain spectrum. In this way, saturated gain should be considered when measuring the modal discrimination.

Supplementary Table 1. The saturated gain of a 400-μm quantum well SOA

Drive Current (mA)	Saturated Output Power	Gain	
		Saturated Gain	Second Mode Gain
17.397	2.3 dBm	0.01 dB	0.50 dB
17.513	2.6 dBm	0.06 dB	0.51 dB
19.500	3.4 dBm	0.99 dB	1.62 dB
19.531	3.4 dBm	1.01 dB	1.64 dB
21.051	4.0 dBm	1.65 dB	2.17 dB
21.422	4.1 dBm	1.81 dB	2.35 dB
21.900	4.1 dBm	2.00 dB	2.61 dB
22.137	4.1 dBm	2.10 dB	2.65 dB
40.293	10.1 dBm	7.28 dB	7.83 dB
40.819	10.1 dBm	7.34 dB	7.88 dB

Supplementary Table II. The lasing conditions for a parity-time-symmetric laser and a single-ring laser

Component	Parity-time-symmetric laser		Single-ring laser	
	Fundamental mode	Next lasing mode	Fundamental mode	Next lasing mode
SOA1	0	0	0	0
SOA2	25.000 mA (~3.7 dB)	25.000 mA (~3.7 dB)	25.000 mA (~3.7 dB)	25.000 mA (~3.7 dB)
SOA3	21.422 mA (~1.81 dB)	40.819 mA (~**7.88** dB)	19.531 mA (~1.01 dB)	21.900 mA (~**2.61** dB)
SOA4	21.051 mA (~1.65 dB)	40.293 mA (~**7.83** dB)	19.500 mA (~0.99 dB)	22.137 mA (~**2.65** dB)
SOA5	17.513 mA (~0.06 dB)	17.513 mA (~0.51 dB)	N/A	N/A
SOA6	17.397 mA (~0.01 dB)	17.397 mA (~0.50 dB)	N/A	N/A
PM1	0	0	0	0
PM2	1.282 mA	1.282 mA	1.282 mA	1.282 mA
PM3	0	0	0	0
PM4	0	0	0	0
PM5	1.282 mA	1.282 mA	2.440 mA	2.440 mA
PM6	0.103 mA	0.103 mA	N/A	N/A
Modal discrimination	~13.19 dB		~3.26 dB	

The saturated gain of an SOA (a 400-µm quantum well SOA) is measured. To do so, we set the injection current at a given value and then increase the input optical power. The saturation power is usually defined as the output power at which the device gain drops by 3 dB from its unsaturated value for a given injection current (5). However, the SOAs are working under small injection currents with a small-signal gain less than 3 dB in our proposed PT-symmetric laser, the saturation power here is defined as the output power at which the device gain drops by 0.5 dB from its unsaturated value for a particular current.

Supplementary Table 1 shows the saturation powers of the SOA at eight given injection currents, which are the currents used to enable the device to operate as a PT-symmetric laser and a conventional single-ring laser, as shown in Supplementary Table 2. In the measurements, a continuous wave (CW) light wave with a wavelength of 1553.8 nm, which is the lasing wavelength of the proposed PT-symmetric laser, is coupled into a standalone 400-µm SOA. At eight given injection currents, by increasing the input optical power, the saturated output powers are measured. To measure the optical gain for a second lasing mode in the SOA, an additional CW light wave with a wavelength of 1553.6 nm and a power of -10 dBm is coupled into the SOA. By adjusting the input power of the first light wave at 1553.8 nm to make the SOA work in saturation for each of the eight given currents, at which the real optical gain for the second light wave at 1553.6 nm is measured, as shown in Supplementary Table 1. The measured optical gain

for the second CW light wave could be used as a reference to estimate the total gain for the second lasing mode, given in Supplementary Table 2. After taking into consideration the gain saturation, the modal discrimination of the proposed PT-symmetric laser is ~13.19 dB, which provides an increased modal discrimination of ~9.93 dB as compared with a conventional single-ring laser.

Supplementary References:

1. Guo, A. *et al.* Observation of PT-symmetry breaking in complex optical potentials. *Phys. Rev. Lett.*, **103**, 093902-4 (2009).
2. Hodaei, H., Miri, M.-A., Heinrich, M., Christodoulides, D. N. & Khajavikhan, M. Parity-time-symmetric microring lasers. *Science*, **346**, 975-978 (2014).
3. Zhu, Y., Zhao, Y., Fan, J. & Zhu, L. Modal gain analysis of Parity-Time-symmetric distributed feedback lasers. *IEEE J. Selected Topics in Quantum Electron.*, **22**, 1500207 (2016).
4. Bisson, J.-F., Lyndin, N., Ueda, K. & Parriaux, O. Enhancement of the mode area and modal discrimination of microchip lasers using angularly selective mirrors. *IEEE J. Quantum Electron.*, **44**, 628-637 (2008).
5. Kim, I., Uppal, K. & Dapkus, P. D. Gain saturation in traveling-wave semiconductor optical amplifiers. *IEEE J. Quantum Electron.*, **34**, 1949-1952 (1998).
